# Does anti-p53 antibody status predict for clinical outcomes in metastatic colorectal cancer patients treated with fluoropyrimidine, oxaliplatin, plus bevacizumab as first-line chemotherapy?

**DOI:** 10.1186/s12885-015-1751-6

**Published:** 2015-10-21

**Authors:** Hiroki Osumi, Eiji Shinozaki, Mitsukuni Suenaga, Yosuke Kumekawa, Mariko Ogura, Masato Ozaka, Satoshi Matsusaka, Keisho Chin, Noriko Yamamoto, Nobuyuki Mizunuma

**Affiliations:** 1 Department of Gastroenterology, The Cancer Institute Hospital, Japanese Foundation for Cancer Research, 3-8-31 Ariake, Koto-ku, Tokyo, 135-8550 Japan; 2 Medical Department of Oncology, The Cancer Institute Hospital, Japanese Foundation for Cancer Research, Tokyo, Japan; 3 Department of Pathology, The Cancer Institute Hospital, Japanese Foundation for Cancer Research, Tokyo, Japan

**Keywords:** Anti-p53 antibody, *KRAS*, Metastatic colorectal cancer, First-line chemotherapy

## Abstract

**Background:**

*TP53* gene mutation is widely known as one of the determinants of impaired chemosensitivity. p53 is a tumor-suppressor protein in humans encoded by the *TP53* gene. Some studies have shown that *TP53* gene mutation and accumulation of the p53 protein are closely related with serum anti-p53 antibody positivity. This study aimed to evaluate the predictive significance of the serum p53 antibody status in metastatic colorectal cancer (mCRC) patients treated with fluoropyrimidine, oxaliplatin, plus bevacizumab as first-line chemotherapy.

**Methods:**

Ninety patients treated with fluoropyrimidine, oxaliplatin plus bevacizumab as first-line chemotherapy were enrolled, including 70 whose *KRAS* genotype was revealed at the beginning of treatment. Before chemotherapy initiation, the serum p53 antibody level was quantified by enzyme-linked immunosorbent assay using MESACUP® anti-p53 test kits. The cutoff value for positivity was 1.3 U/mL, as calculated previously. The *KRAS* genotype of the tumor samples was analyzed using the Luminex® assay.

**Results:**

Overall response rates of Response Evaluation Criteria in Solid Tumors criteria were 77.7 % (42/54) in anti-p53–negative patients and 69.4 % (25/36) in anti-p53–positive patients. The odds ratio was 1.07. Median overall survival was 36.1 months in the anti-p53–positive patients, and not available in the anti-p53–negative patients (hazard ratio, 0.81; 95 % confidence interval, 0.37–1.77; *P* = 0.61). The corresponding values for median progression-free survival were 13.3 months and 14.6 months (hazard ratio, 0.69; 95 % confidence interval, 0.41–1.17; *P* = 0.17), respectively.

**Conclusions:**

Serum anti-p53 antibody positivity did not predict chemoresistance in mCRC treated with fluoropyrimidine, oxaliplatin, plus bevacizumab as first-line chemotherapy.

## Background

In 1988, Vogelstein et al. proposed a multistage theory of carcinogenesis known as the adenoma–carcinoma sequence, in which colorectal cancer (CRC) arises because of mutations that activate multiple oncogenes and inactivate tumor-suppressor genes. These mutations accumulate in the normal colonic epithelial cells and cause adenomas. *TP53* mutations were proposed as the driver mutations in colorectal carcinogenesis [1].

Furthermore, the *TP53* gene mutation is widely known as an important determinant of impaired chemosensitivity [2]. Approximately 40–50 % of CRC lesions are reported to carry either a mutation in *TP53* and/or loss of a heterozygote at chromosome 17q, where *TP53* is located [3]. Several in vitro studies have reported a relationship between *TP53* mutation status and sensitivity to a number of cytotoxic agents, including fluoropyrimidines [4]. Furthermore, the presence of a *TP53* mutation in tumors is associated with shorter patient survival compared with the presence of wild-type *TP53*.

p53 is a tumor-suppressor protein encoded by the *TP53* gene in humans. Mutations commonly result in expression of proteins with abnormal conformation, which is readily detected as a p53 overexpression by immunohistochemistry (IHC). Furthermore, p53 is crucially involved in the control of the cell cycle and apoptosis and is also frequently altered in CRC. Some studies have shown that *TP53* gene mutation and accumulation of the p53 protein are closely related with the presence of serum anti-p53 antibodies [5]. Anti-p53 antibodies are independent prognostic factors in esophageal and ovarian cancer patients treated with chemotherapy [6]. Thus, the presence of serum p53 antibodies could theoretically predict chemoresistance in metastatic CRC (mCRC) treated with chemotherapy. However, no reports showed about the relationship between anti-p53 antibody and chemosensitivity in mCRC patients.

On the other hand, potential biomarkers include mutations in *KRAS* and *BRAF*, which result in constitutive signaling through the oncogenic *Ras/Raf/MEK/ERK* pathway. Patients carrying tumors with *KRAS* mutations are also reported to have a poorer prognosis. For example, *TP53* mutation in combination with *KRAS* mutation at codon 13 are associated with a worse prognosis in CRC [7]. However, no reports showed about the relationship between anti-p53 antibody and *KRAS* mutation.

Therefore, we investigated the relationship between anti-p53 antibody and *KRAS* genotype and whether the anti-p53 antibody status, IHC of p53 protein status and *KRAS* genotype are correlated to chemosensitivity and prognostic factors such as overall survival (OS) and progression-free survival (PFS) in mCRC patients treated with fluoropyrimidine, oxaliplatin, plus bevacizumab as first-line chemotherapy.

## Methods

This study has been performed in accordance with the Declaration of Helsinki. The cancer Institute Hospital of Japanese Foundation for Cancer Research, Institutional Review Board approved this study (Registry number: 1278). We obtained a comprehensive written informed consent about the research before chemotherapy was started.

### Study population

We enrolled 90 patients who confirmed mCRC and received first-line chemotherapy (FOLFOX or XELOX with Bev) at the Cancer Institute Hospital between January 2009 and November 2010, and measured anti-p53 antibody before receiving first-line chemotherapy.

### Treatment and follow-up

The FOLFOX regimen was administered as follows: oxaliplatin on day 1 at a dose of 85 mg/m^2^ as a 2-h infusion concurrent with levofolinic acid at 200 mg/m^2^/day, followed by bolus 5-fluorouracil (5-FU) at 400 mg/m^2^ and a 22-h infusion of 5-FU at 2400 mg/m^2^ for 2 consecutive days. Bevacizumab was administered at a dose of 5 mg/kg in a 30-min intravenous infusion on day 1 in 2-week cycles.

The XELOX regimen was administered as follows: capecitabine (2000 mg/m^2^, biweekly) plus oxaliplatin (130 mg/m^2^, day 1). Bevacizumab was administered at a dose of 7.5 mg/kg in a 30-min intravenous infusion on day 1 in 3-week cycles.

The treatment was repeated every 2 (or 3) weeks until disease progression or unacceptable toxicity occurred, or until a patient chose to discontinue treatment.

In our hospital, the patients underwent computed tomography scans approximately every 3 months after treatment completion and were regularly assessed for response to chemotherapy and local or distant recurrence. The evaluation was repeated every 3 (or 4) courses, or more frequently in patients with clinically suspected progression. In this study, tumor response was reassessed via computed tomography using the Response Evaluation Criteria in Solid Tumors (RECIST), version 1.1.

### Enzyme Immunoassay for p53antibody, IHC of p53 protein and KRAS genotyping

The serum anti-p53 antibody status was evaluated in each patient before initiation of first-line chemotherapy. The evaluation was performed by enzyme-linked immunosorbent assay (ELISA) using the anti-p53 ELISA Kit (MESACUP, Nagoya, Japan). This kits have been developed with less variation in seropositivity (13–27 %) with intra- and inter-assay coefficient of variation of 1.85–2.37 % and 0.3–3.32 % respectively [8]. For anti-p53 autoantibodies, the cut off for positivity was set at the average value among healthy subjects plus 3 standard deviations or plus 1 standard deviation. The cut-off value for positivity was calculated as 1.3 U/mL, as reported previously [2]. In addition, immunostaining was performed with anti p53 protein antibody (D0-7,DAKO, Glostrup, Denmark) on formalin-fixed paraffin-embedded fragments obtained from those patients from whom adequate tissue samples could be obtained by biopsy or surgical resection. Nuclear staining of tumor cells were judged as positive for p53 protein. The percentage of p53 positive cancer cells was calculated compared with HE staining. The positive rate of ≥ 70 % was determined as overexpression of p53 protein. The *KRAS* genotype of the tumor samples was analyzed using the Luminex assay, as previously reported [8]. The sensitivity of KRAS testing by Luminex has been reported to be 10 % [9].

### Statistical analysis

Percentages were compared using the chi-square or Fisher’s exact test when appropriate. Quantitative variables were compared using Student’s *t* test. Follow-up was estimated using the Kaplan-Meier method. The correlation between p53 antibody and the *KRAS* genotype, IHC of p53 protein were estimated using Pearson’s correlation coefficient. OS and PFS were estimated using the Kaplan-Meier method and compared using the log-rank test. PFS was defined as the interval beginning with chemotherapy to relapse or death, whichever occurred first. Variables associated with OS or PFS with a *P* value <0.2 in a univariate analysis were included in a multivariate ascending stepwise Cox regression analysis. In the Cox model, continuous variables were dichotomized. All statistical analyses were performed with EZR (Saitama Medical Center, Jichi Medical University), which is a graphical user interface for R (The R Foundation for Statistical Computing). All reported *P* values were two-sided, and *P* values <0.05 were considered significant.

## Results

### Patients characteristics (Table 1)

**Table 1:** Patients characteristics

	ITT popuration (*n* = 90)		KRAS wild type		KRAS mutant	
	p53 antibody		p53 antibody		p53 antibody	
	Positive	Negative	Positive	Negative	Positive	Negative
	(*n* = 36)	(*n* = 54)	(*n* = 11)	(*n* = 31)	(*n* = 13)	(*n* = 13)
Gender, n (%)						
Male	25(69.4)	30(55.5)	9(81.8)	16(51.6)	9(69.2)	8(61.5)
Female	11(30.5)	24(44.4)	2(18.1)	15(48.3)	4(30.7)	5(38.4)
Age						
Median (range)	58.4(39–74)	60.9(39–75)	57.3(41–73)	59.8(39–71)	59.3(39–74)	61.3 (41–75)
<65, n (%)	26(72.2)	31(59.2)	8(72.7)	19(61.2)	9(69.2)	6(46.1)
≧ 65, n (%)	10(27.7)	23(42.5)	3(27.2)	12(38.7)	4(30.7)	7(53.8)
ECOG PS at base line, n (%)						
	0 33(91.6)	51(94.4)	11(100)	30(96.7)	9(69.2)	12(92.3)
	1 3(8.3)	3(5.5)	0(0)	1(3.1)	3(23.0)	1(7.7)
Liver metastasis, n (%) 12(33.3)		31(57.4)	7(63.6)	19(61.2)	3(23.0)	5(38.4)
Lung metastasis, n (%) 12(33.3)		22(40.7)	5(45.4)	9(29.0)	4(30.7)	8(61.5)
Lymph node metastasis, n (%)	21(66.6)	26(48.1)	8(72.7)	17(54.8)	8(61.5)	4(30.7)

*ITT* intention to treat, *PS* performance status

**Table 2:** Clinical response after 1st line chemotherapy

	ITT popuration (*n* = 90)		KRAS wild type		KRAS mutant	
	p53 antibody		p53 antibody		p53 antibody	
	Positive	Negative	Positive	Negative	Positive	Negative
n (%)	*n* = 36	(*n* = 54)	(*n* = 11)	(*n* = 31)	(*n* = 13)	(*n* = 13)
Complete Response	5(13.8)	2(3.7)	1(9)	1(3.2)	3(23)	0(0)
Partial Response (PR)	21(58.3)	41(74.0)	9(81.8)	25(80.6)	7(53.8)	9(69.2)
Stable Disease	5(5.5)	10(18.5)	1(9)	3(9.7)	3(23)	4(30.7)
Progressive Disease	2(8.3)	1(1.8)	0(0)	1(3.2)	0(0)	0(0)
Not Evaluable	3(8.3)	0(0)	0(0)	1(3.2)	0(0)	0(0)
PR in	26(72.2)	43(79.6)	10(91)	26(83.8)	10(76.9)	9(69.2)
Odds ratio (95 % CI)	1.1 (0.55–2.21)		0.92 (0.3–2.8)		0.9 (0.23–3.43)	
*P* value	0.87		1		1	

Between January 2009 and November 2010, 90 patients were referred for first-line chemotherapy for mCRC. Median age of the patients at the time of measuring anti-p53 antibody was 61 years old (±9.1). This cohort was composed of males (38.8 %) and females (61.2 %). Serum anti-p53 antibodies were detected in 40.0 % patients (36/90). IHC analyzed with monocloncal antibodies against p53 of the patients was detected in 63 % (38/60). There was no significant difference in background between the anti-p53–positive and anti-p53–negative groups.

### Correlation between anti-p53 antibody status and clinical outcomes (*n* = 90)

**Fig. 1:**
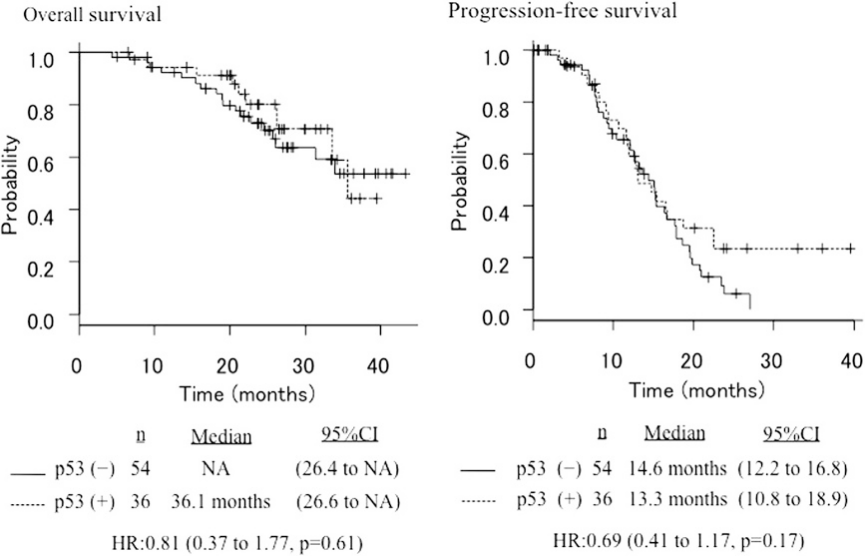
Overall survival and progression-free survival curves according to p53 antibody status for the entire population (*n* = 90)

Applying RECIST criteria (Table 2), the overall response rate (ORR) was 77.7 % (42/54) in the anti-p53–negative patients and 69.4 % (25/36) in the anti-p53*–*positive patients. The odds ratio was 1.07. Median OS was 36.1 months in the anti-p53–positive patients and not available (NA) in the anti-p53–negative patients [hazard ratio (HR) 0.81, 95 % confidence interval (CI) 0.37–1.77, *P* = 0.61]. The corresponding values for median PFS were 13.3 months and 14.6 months (HR, 0.69; 95 % CI, 0.41–1.17; *P* = 0.17), respectively (Fig. 1).

### Correlation between IHC of p53 protein status and clinical outcomes (*n* = 60)

**Fig. 2:**
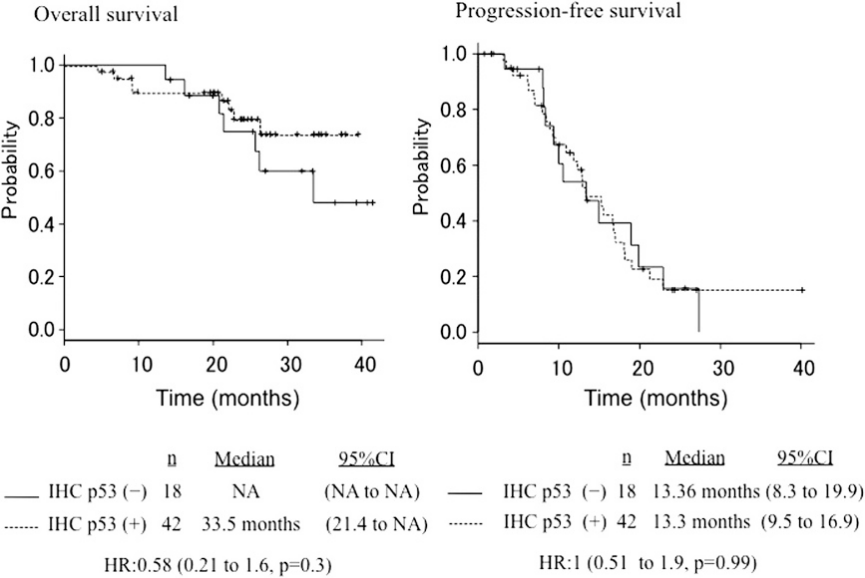
Overall survival and progression-free survival curves according to IHC of p53 protein status (*n* = 60)

ORR according to RECIST criteria was 77.7 % (14/18) and 76.1 % (32/42) in the p53 protein negative tumors and the p53 protein positive tumors, respectively. The odds ratio was 1.09.OS was 33.5 months in the p53 protein negative tumors, and NA in the p53 protein positive tumors (HR 0.58, 95 % CI 0.21-1.6, *P* = 0.3). PFS was 13.36 months, and 13.3 months (Table 2), respectively (HR 1.0, 95 % CI 0.51-1.9, *P* = 0.99) (Fig. 2). The estimated correlation between anti-p53 antibody positivity and the IHCof p53 protein positive tumors was 0.32 (95 % CI 0.07–0.53, *P* = 0.012).

### Correlation between anti-p53 antibody status and *KRAS* genotype (*n* = 70) (Table 2)

**Fig. 3:**
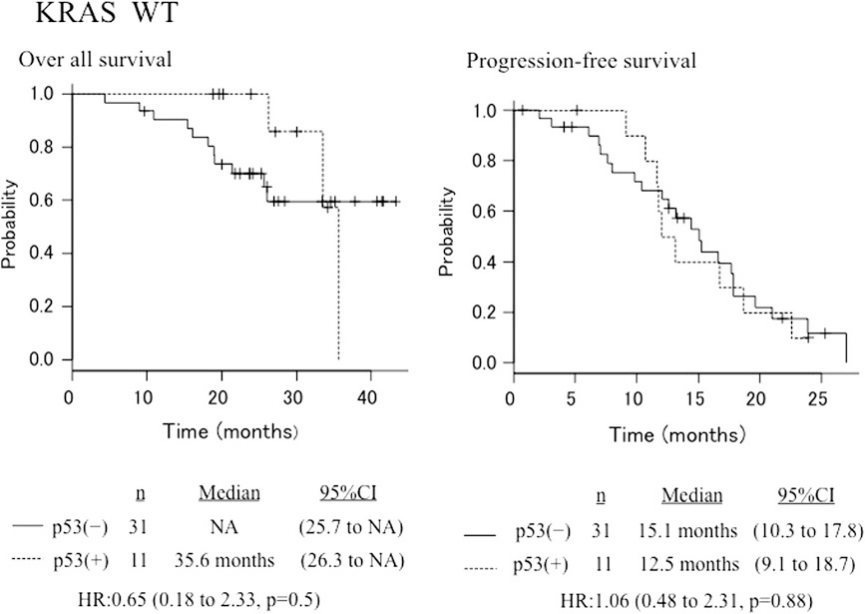
Overall survival and progression-free survival curves according to p53 antibody status for the KRAS wild-type population (*n* = 44)

**Fig. 4:**
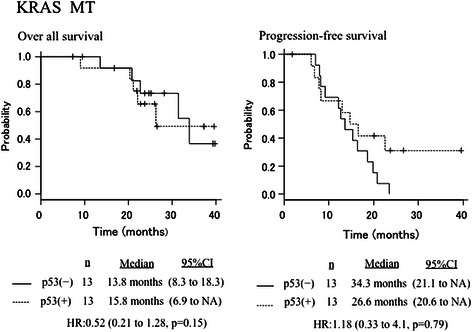
Overall survival and progression-free survival curves according to p53 antibody status for the KRAS mutant-type population (*n* = 26)

In the KRAS wild-type (*n* = 42) patients, ORR according to RECIST was 90.9 % and 83.8 % in the anti-p53–negative patients and anti-p53–positive patients, respectively. Median OS was 35.6 months in all patients, 35.6 months in the anti-p53–negative patients, and NA in the anti-p53–positive patients (HR 0.65, 95 % CI 0.18–2.33, *P* = 0.5). The corresponding values for median PFS were 14.6 months in total, 17.9 months, and 16.7 months, respectively (HR 1.06, 95 % CI 0.48–2.31, *P* = 0.88) (Fig. 3). In the KRAS mutant-type (*n* = 26) patients, ORR according to RECIST was 69.2 % (9/13) and 76.9 % (10/13) in the anti-p53–negative patients and anti-p53–positive patients, respectively. Median OS was 33.8 months in all patients, 13.8 months in the anti-p53–negative patients, and 15.8 months in the anti-p53–positive patients (HR 0.52, 95 % CI 0.21–1.28, *P* = 0.15). The corresponding values for median PFS were 14.6 months, 34.3 months, and 26.6 months, respectively (HR 1.18, 95 % CI 0.33–4.1, *P* = 0.79) (Fig. 4). The estimated correlation between anti-p53 antibody positivity and the KRAS genotype was 0.037 (95 % CI 0.20–0.27, *P* = 0.746).

### Univariate and murtivariate analysis

In univariate analysis, peritoneal metastasis and multiple metastasis were also significant predictors of OS. On the other hand, lung metastasis and lymph node metastasis were also significant predictors of PFS. In the multivariate analysis, peritoneal metastasis was significant predictors of OS and lung metastasis and lymph node metastasis were significant predictors of PFS. However both anti-p53 antibody and IHC of p53 protein negativity did not yield any independent predictive factors (Table 3).

**Table 3:** Univariate and multivariate analysis

Univariate analysis				
OS	HR	Lower 95 % CI	Upper 95 % CI	*p*.value
Gender (male or female)	0.76	0.34	1.67	0.5
Age (<65 or 2^ 65)	0.98	0.94	1.03	0.5
Performance status (0 or 1)	1.78	0.42	7.5	0.43
Resection of primary tumor (yes or no)	0.7	0.2	1.6	0.43
Ascitis (yes or no)	1.7	0.7	4.1	0.22
Liver metastasis (yes or no)	1.2	0.59	2.6	0.55
Lung metastasis (yes or no)	0.77	0.34	1.7	0.51
Lymph metastasis (yes or no)	1.9	0.86	4.2	0.1
Multiple metastasis (yes or no)	2.5	1.1	5.9	0.03
Peritoneal metastasis (yes or no)	2.5	1.2	5.2	0.01
Anti p53 antibody (positive or negative)	0.8	0.3	1.7	0.61
IHC of p53 protein (positive or negative)	0.58	0.21	1.6	0.3
KRAS (wild or mutant)	1.29	0.54	2.75	0.63
PFS				
Gender (male or female)	1.6	0.9	3.03	0.1
Age (<65 or^65)	0.99	0.96	1.02	0.53
Performance status (0 or 1)	1.8	0.23	2.47	0.64
Resection of primary tumor (yes or no)	1.5	0.72	3.1	0.99
Ascitis (yes or no)	0.6	0.28	1.43	0.29
Liver metastasis (yes or no)	0.54	0.28	1	0.07
Lung metastasis (yes or no)	2.8	1.59	5.2	4E-04
Lymph metastasis (yes or no)	0.47	0.26	0.85	0.01
Multiple metastasis (yes or no)	0.9	0.51	1.6	0.72
Peritoneal metastasis (yes or no)	0.73	0.37	1.45	0.27
Anti p53 antibody (positive or negative)	0.9	0.49	1.6	0.73
IHC of p53 protein (positive or negative)	1	0.51	1.9	0.99
KRAS (wild or mutant)	0.98	0.49	1.9	0.94
Multivariate analysis				
OS	HR	Lower 95 % CI	Upper 95 % CI	*p*.value
Peritoneal metastasis (yes or no)	2.3	1.1	5.1	0.02
PFS				
Lung metastasis (yes or no)	2.46	1.34	4.51	0.003
Lymph metastasis (yes or no)	0.5	0.28	0.97	0.04

## Discussion

To our knowledge, this retrospective study is the first to evaluate the predictive significance of the presence of anti-p53 antibodies and its correlation with the *KRAS* genotype in CRC patients treated with first-line chemotherapy. No correlation was observed between anti-p53 antibody positivity and ORR. Furthermore, no correlation was observed between anti-p53 antibody positivity and the *KRAS* genotype.

The mechanism underlying anti-p53 auto-antibody production has yet to be revealed but is thought to be associated with the presence of the *TP53* mutation and p53 protein overexpression. Anti-p53 autoantibody frequency was then correlated with reported *TP53* mutation rates to determine the association between anti-p53 antibody positivity and the *TP53* mutation status (CRC: *TP53* mutation 43.3 %, anti p53 antibody positivity 21.4 %). Moderate correlation (r^2^ = 0.45, correlation 0.59) was found to exist between anti-p53 antibody positivity and the *TP53* mutation [10]. Mutational loss of the tumor-suppressor functions of *TP53* has been associated with decreased sensitivity to agents inhibiting DNA synthesis, such as 5-FU [11]. These genetic alterations play crucial roles in colorectal carcinogenesis, including DNA damage signaling and the response to platinum-based chemotherapeutic agents.

As mentioned above, preclinical research has indicated that disruptions in the Ras/Raf/MEK/ERK pathway or inactivation of the *TP53* tumor-suppressor gene may have clinical relevance to the efficacy of anti-VEGF agents, such as bevacizumab.

However, in this retrospective study, we did not assess mCRC patients who were more likely to respond to bevacizumab therapy.

There are some reasons to explain the results of this study. First, An Anti-p53 antibody is not normally produced wild type p53 protein induces tolerance of the host. However *TP53* mutation alone is insufficient to trigger anti-p53 antibody production. Only 20–50 % of patients which detectable *TP53* mutations produce detectable anti-p53 antibodies [12]. This is attributed to the type of mutation, mis-sense mutations is associated with higher antibody production compared with other mutation [13]. Second anti-p53 antibodies most frequency recognize terminal epitopes but not the central domain with majority of the mutation [8]. Third, the differences in individual’s immune systems might relate, the humoral response is independent on the individual’s MHC presentations [8].

The methods used to determine the mutational status of *TP53* or *KRAS* merit discussion. Indeed, the question is whether anti-p53 antibodies are a reliable parameter for the *TP 53* mutation status. These antibodies have high specificity but lack sensitivity [4]. They have the same drawbacks as immunohistochemistry because they are absent in patients in whom *TP53* mutations negate p53 protein synthesis and accumulation.

In this study we also investigate whether IHC of p53 protein was the predictive factor of chemosensitivity or not, however there was no relationship between IHC of p53 protein and clinical outcomes.

Other techniques, such as sequencing and functional assays, have been developed to determine the mutation status of *TP53* as it applies to CRC. In previous studies, perioperative variations in serum anti-p53 antibody levels have been shown to predict OS (Table 4) [12, 14–24]. However, only the sequencing data were correlated with the level of chemoresistance (Table 5) [4, 11, 25–31]. Anti-p53 antibody has low sensitivity in CRC but is nearly 100 % specific for malignancy. Thus, we believe anti-p53 antibody measurement is suitable and cost-effective for screening a high-risk population and for postoperative cancer surveillance as a guide for earlier detection of recurrence [29].

**Table 4:** p53 status and prognosis of colorectal cancer: past literature date

Reference	n	Histology treatment		Methods for determing p53 Ab	IHC	Sequencing	Frequency alterd p53 pathway (%)	Prognostic value		
								Overall survial	Survival	Response
LAN YT [10]	258	ACC	surgery	-	+	-	37.6(IHC)	univariate	NA	NA
Triantafyllou K [11]	55	Adenoma	Polypectomy	-	+	-	41.8DtHCD	NA	murtivariate	NA
Wang Q [12]	40	ACC	surgery	-	+	-	65 OHCD	univariate	NA	NA
Hu J [13]	120	ACC	biopsy and surgery	-	+	-	57 OHCD	univariate	NA	NA
Grewal H [14]	66	ACC	surgery	-	+	-	51.5(IHC)	NS	NA	NA
Bouzourenne H [15]	122	ACC	surgery	-	+	+	47(IHC)	univariate	NA	NA
							32(S)	murtivariate		
Samowitz WS [16]	1464	ACC	biopsy and surgery	-	-	+	45.4DSD	univariate	NA	NA
Chang SC [17]	167	ACC	surgery	+	-	+	28.1(Ab)	univariate	NA	NA
							56.3(S)	murtivariate		
Angelopoulou K [18] 229		ACC	biopsy and surgery	+	-	-	23(Ab)	NS	NS	NA
Kressner U [19]	184	ACC	surgery	+	-	-	32(Ab)	univariate	NA	NA
Suppiah A [20]	28	ACC	surgery	+	-	-	21,7(Ab)	NS	NS	NA
Kreessner U [21]	294	ACC	biopsy	+	-	-	55DAbD	NS	NA	NA

*Ab* antibody; *IHC* immunohistochemistry; *S* sequencing; *ACC* advanced colorectal cancer; *NA* not available; *NS* not significant

**Table 5:** p53 status and prognosis of colorectal cancer: comparison between literature deta and the present report

Reference	n	Histology treatment		Methods for determing p53 Ab	IHC	Sequencing	Frequency of alterd p53 pathway (%)	Prognostic value	Event-free survival	Response
								Overall survial		
Popat S [22]	967	CRC	Adjuvant	-	+	-	60 (IHC)	NS	NA	NA
Zaana A [23]	233	CRC	Adjuvant	-	+	-	53 (IHC)	NA	NS	NA
Ahn MJ [24]	45	mCRC	chemotherapy	-	+	-	80 (IHC)	NA	NA	NS
Berglund A [25]	122	mCRC	chemotherapy	-	+	-	60 (IHC)	NS	NA	NS
Ince WL [26]	295	CRC	chemotherapy	-	+	+	68 (IHC), 72(S)	NS, NS	NA	NA
Mollevi DG [27]	91	mCRC	chemotherapy	-	-	+	50.5 (S)	multivariate	NA	NA
Rosty C [28]	56	mCRC	chemotherapy	-	-	+	62.5 (S)	univariate	NA	NS
Westra JL [29]	220	CRC	Adjuvant	-	-	+	53(S)	NA	murtivariate	NA
Oden-Gangloff [30]	64	mCRC	chemotherapy	-	-	+	64(S)	NA	murtivariate	NA
Present study	90	mCRC	chemotharapy	+	+	-	40(Ab), 63(IHC)	NS,NS	NS,NS	NS,NS

*Ab* antibody; *IHC* immunohistochemistry; *S* sequencing; *(m)CRC* (metastatic) colorectal cancer; *NA* not available; *NS* not significant

This study had some limitations. Because of its retrospective and single-center nature, an unknown bias may exist in the findings. Furthermore, we didn’t measure *TP53* mutation using sequencing method which is one of the main methods of detect *TP53* mutation. When we assess the relationship between *TP53* gene mutation and chemoresistance in mCRC patients, we should use other methodologies such as sequencing and functional assays, apart from the anti-p53 antibody status.

## Conclusion

Serum anti-p53 antibody positivity did not predict chemoresistance in mCRC treated with fluoropyrimidine, oxaliplatin, plus bevacizumab at first-line chemotherapy. We believe that if we want to know the relationship between the anti-p53 antibody status and chemosensitivity, we should use other methodologies like sequencing, and functional assays, apart from the anti-p53 antibody status.

## Abbreviations

(m)CRC: Metastatic colorectal cancer
IHC: Immunohistochemistry
KRAS: v-Ki-ras2 Kirsten rat sarcoma viral oncogene homolog
BRAF: v-Raf murine sarcoma viral oncogene homolog B
MEK: Mitogen-activated protein kinase kinase
ERK: Extracellular signal-regulated kinase
OS: Overall survival
PFS: Progression free survival
Bev: Bevacizumab
RESIST: Response evaluation criteria in solid tumors
ELISA: Enzyme-linked immuno- sorbent assay
ORR: Overall response rate
NA: Not available
HR: Hazard ratio
CI: Confidence Interval
VEGF: Vascular endothelial growth factor
MHC: Major histocompatibility complex
